# Hypothermia and advanced neuromonitoring

**DOI:** 10.1186/cc11285

**Published:** 2012-06-07

**Authors:** Raimund Helbok, Alois Schiefecker, Marlene Fischer, Anelia Dietmann, Erich Schmutzhard

**Affiliations:** 1 Department of Neurology, Neurocritical Care Unit, Medical University Hospital Innsbruck, Austria

## 

Therapeutic hypothermia (TH) improves short-term neurologic outcome and reduces mortality after cardiac arrest (CA) [1]. Neuroprotective mechanisms comprise reduced cerebral metabolic demand [2,3], decreased excitotoxicity [4-7], cell membrane stabilization [8,9], inhibition of spreading depolarizations [10] and cytokine release [11], and preservation of cerebral autoregulation. This concept of neuroprotection led to clinical trials using TH for the prevention of secondary brain injury in patients with traumatic brain injury, subarachnoid hemorrhage (SAH) and ischemic stroke; however, they failed to show any benefit in clinical outcomes [12-15]. This may reflect our lack of understanding of the exact pathophysiologic processes induced by TH and the potential harm through hypothermia and rewarming injury [16].

Advanced neuromonitoring techniques allow online measurement of brain metabolism, cerebral autoregulation, brain tissue oxygenation, and cerebral blood flow (CBF) and provide information about brain function, energy supply and demand [17-19]. Here we will summarize current knowledge about the effects of TH on brain homeostasis after acute brain injury using advanced neuromonitoring techniques and call for more observational trials investigating pathophysiologic mechanisms during TH and rewarming to maximize the benefits of this emerging therapeutic modality.

TH effectively decreases intracranial pressure (ICP) by up to 10 mmHg [12,20-22], whereby mild hypothermia (35°C) seems to be as effective as 33°C [21,23]. Mechanisms include the reduction in metabolic demand and the inhibitory effect on inflammation and free radical production stabilizing the blood-brain barrier and decreasing vasogenic edema [20,24-26].

Cerebral microdialysis allows bedside monitoring of cerebral metabolic changes from the extracellular fluid of the brain [27]. Decreased energy supply, increased demand or mitochondrial dysfunction may result in brain metabolic distress and/or brain hypoglycemia (brain glucose <0.7 mmol/l). Cerebral microdialysis is feasible during TH and sensitive to detect secondary energy failure as indicated by an increase in the lactate-pyruvate ratio (LPR) [28,29]. Therapeutic hypothermia reduces brain metabolic demand for oxygen and glucose and preserves ATP supply to the brain decreasing the risk of secondary energy failure [29-32]. Moreover, extensive cerebral lactate accumulation is inhibited by TH, which ameliorates the deleterious effects on cell membranes and the blood-brain barrier [33]. Increased brain glucose may be found during TH [6]; however, an increased blood glucose variability during hypothermia has been reported [34]. This may negatively affect brain glucose as sudden decreases in systemic glucose have been associated with brain metabolic distress and worse outcome [35]. Therefore monitoring of brain glucose is important to detect brain hypoglycemia and prevent further neuronal damage during TH and the rewarming phase. An additional beneficial effect of hypothermia that has been extensively studied *in vitro *and in animal models is the reduction in excitotoxic neurotransmitter release [4-7,28], thereby inhibiting nitric oxide synthesis and apoptotic pathways [16,36]. A decrease of extracellular glutamate was observed during TH after cardiac arrest and in ischemic stroke patients using cerebral microdialysis [28,37]. In summary, cerebral microdialysis allows monitoring of the metabolic effects of TH after acute brain injury.

Another mechanism how TH may reduce secondary energy failure is a decrease in oxygen consumption through diminished metabolism [2,3], resulting in increased brain tissue oxygenation [25,38]. Brain tissue oxygenation reflects the net effect of oxygen delivery, diffusion and consumption and can be assessed by positron emission tomography, magnetic resonance spectroscopy, near-infrared spectroscopy or by invasive P_b_tO_2 _probes [39,40]. Therapeutic hypothermia below 35°C may impair brain tissue oxygenation through a left-shift of the oxygen dissociation curve, therefore enhancing the affinity of oxygen to hemoglobin, or by decreasing delivery of oxygen to the brain [21,41]. Jugular bulb oxygen saturation (jSvO_2_) is a global measurement of brain oxygen extraction and is increased during mild TH [31,41], reflecting a reduction in cerebral metabolic rate of oxygen.

Shivering is frequently observed during TH and may abolish the neuroprotective effect of temperature modulation through increase in metabolic demand and systemic and cerebral energy consumption [42]. A shivering-associated reduction in P_b_tO_2 _seems to correlate with the intensity of therapeutic cooling and potentially increases the risk of brain hypoxia [43]. These results imply that the neuroprotective effect of TH may be most beneficial at a temperature not lower than 35°C and shivering should be assessed at the bedside and effectively treated by pharmacological and nonpharmacological means [42].

It is important to note that CO_2 _reactivity may be preserved during TH [44]. Therapeutic hyperventilation as TH is used as rescue therapy to decrease raised ICP and unintentional hypocapnia is also commonly observed in patients with acute brain injury [45], which increases the risk of brain tissue hypoxia [38]. Monitoring of brain tissue oxygen or jugular venous oxygen saturation (jSvO_2_) is recommended for patients with acute traumatic brain injury, where therapeutic hyperventilation is used [46] and is important especially during TH. Preserved cerebral autoregulation seems not to be disturbed during TH and early induction of hypothermia after SAH led to faster restoration of cerebrovascular reactivity *in vivo *[6,47,48].

The rewarming has been considered as the vulnerable phase following TH [42,49,50]. Rapid rewarming and timing in vulnerable phases of the injured brain may abolish the neuroprotective effects of TH through ICP increase, excitotoxicity, increased metabolic demand and derangement of cerebrovascular reactivity [42,51-53]. A report of four patients treated with TH after CA observed an increase in LPR in all patients during rewarming indicating brain ischemia [28]. Slow and controlled rewarming after moderate hypothermia may prevent ICP increase and glutamate release and stabilize infarct volume [53]. Close monitoring of cerebral metabolism, ICP, CBF and brain tissue oxygenation can help to define the optimal rewarming rate to avoid increases in ICP (recommended rate of 0.1°C) and to early detect an imbalance in energy supply and demand.

## Conclusion

In the clinical setting there is still need to further explore the best induction and maintenance method, optimal duration and targeted temperature of therapeutic hypothermia. Due to the complexity of pathophysiologic mechanisms during hypothermia and rewarming, combining different advanced monitoring techniques seems mandatory. Multimodal neuromonitoring guidance may then help to define therapeutic targets and to establish clinical protocols to maximize the benefits of this emerging therapeutic modality.
